# Identify latent chromosomal aberrations relevant to myelodysplastic syndromes

**DOI:** 10.1038/s41598-017-10551-3

**Published:** 2017-09-04

**Authors:** Qibin Song, Yuxin Chu, Yi Yao, Min Peng, Weihong Yang, Xiaoqing Li, Shiang Huang

**Affiliations:** 1 Cancer Center, Renmin Hospital of Wuhan University, Wuhan, China; 2 Molecular department, Kindstar global, Wuhan, China

## Abstract

Myelodysplastic syndromes (MDS) are a group of heterogeneous hematologic malignancies. This study aims to identify latent chromosomal abnormalities relevant to MDS, which may optimize the current diagnosis of MDS. Affymetrix CytoScan 750 K microarray platform was utilized to perform a genome-wide detection of chromosomal aberrations in the bone marrow cells of the patients. The findings were compared with the results from traditional karyotypic analysis and FISH to reveal latent chromosomal aberrations. Chromosomal gain, loss, and UPD, and complex karyotypes were identified in those samples. In addition to established cytogenetic aberrations detected by karyotypic analysis, CytoScan 750 K microarray also detected cryptic chromosomal lesions in MDS. Those latent defects underlying multiple gene mutations may construe the clinical variability of MDS. In Conclusion, Affymetrix CytoScan 750 K microarray is efficient in identifying latent chromosomal aberrations in MDS.

## Introduction

Myelodysplastic syndromes (MDS) constitutes a group of heterogeneous premalignant disorder of clonal hematopoietic stem cells (HSC), typically characterized by hypercellular bone marrow with immature blood cell lineages, leading to ineffective hemopoiesis, dysplasia, peripheral blood cytopenia, and frequent evolution to acute myeloid leukemia (AML)^1^. MDS often affects the elderly patients with a mean age of 70 years, with an incidence of 3-5/100000 persons^2^. Chromosomal abnormalities are frequently found in the bone marrow cells of about 50–60% primary MDS and in 80% of secondary MDS patients^3^. Since chromosomal lesions have a great influence on the diagnosis and prognosis of MDS, it has become clear that precise cytogenetic analysis is vital for an accurate diagnosis of MDS^4^. Common chromosomal aberrations include copy number variation (CNV), acquired uniparental disomy (UPD), and complex karyotypes. Chromosomal gain may engender the amplification of oncogenes. On the contrary, chromosomal loss may lead to deletion of tumor suppressor genes (TSGs)^5^. UPD results from mitotic recombination when segments of homologous chromosomes are exchanged, hence both copies of a chromosome pair that are inherited from one parent. Identification of UPD has important significance for investigating the pathogenesis of MDS^6^. Complex chromosomal aberrations (≥3 aberrations) are found in about 20% of MDS patients and are related to an increased risk of progress into AML with unfavorable prognosis^7^. More importantly, additional previously cryptic chromosomal lesions may affect the phenotypes of well-established aberrations. Those small cryptic aberrations may have diagnostic significance.

Currently, traditional metaphase cytogenetics (MC) still remains a gold standard in karyotype analysis of MDS. However, 40–50% of myelodysplastic syndromes (MDS) patients do not exhibit karyotypic abnormalities that can be detected by classical cytogenetic techniques^8^. Especially, UPD is not recognizable by MC because the chromosome banding patterns remain preserved^6^. Fluorescence *in situ* hybridization (FISH) may complement MC analysis, but its application is confined to identify particular chromosomal lesions by the probes utilized. The genetic complexity of malignant cells implores more precise genome-wide techniques, in order to identify some cryptic chromosomal aberrations in mixed cell lines^9^. The advent of high-resolution single nucleotide polymorphism array (SNP-A) technique has enabled a genome-wide scanning of specific chromosomal abnormalities previously undetectable by conventional MC or FISH^10^.

In this study, we have demonstrated the feasibility of applying Affymetrix Cytoscan 750 K Microarray to identify chromosomal CNV, UPD, and complex karyotypes in MDS patients. We postulate that Affymetrix Cytoscan 750 K Microarray would not only identify established chromosomal defects, but also reveal previously subliminal chromosomal lesions in MDS. Identification of those latent chromosomal aberrations may contribute to the stratification of subtypes in MDS, assign appropriate phenotypes, and design individualized treatment.

## Results

### Clinical features of the patients

Conventional metaphase cytogenetic assay, FISH and Affymetrix Cytoscan 750 K Microarray were utilized to detect the common and latent chromosomal lesions for the patients. We selected 25 representative patients for our study, including 17 male and 8 female. Their ages range from 4-86 years old. 10 patients with CNV, 10 patients with UPD, and 5 patients with complex karyotypes were typically presented. The cohort comprises patients with RA(n = 4), RARS(n = 3), RCMD(n = 3), RCMD-RS(n = 1), RAEB-1(n = 2), RAEB-2(n = 2), 5q- syndrome(n = 1), MDS-U (n = 1), sAML(n = 8).

### CNV

**Figure 1:**
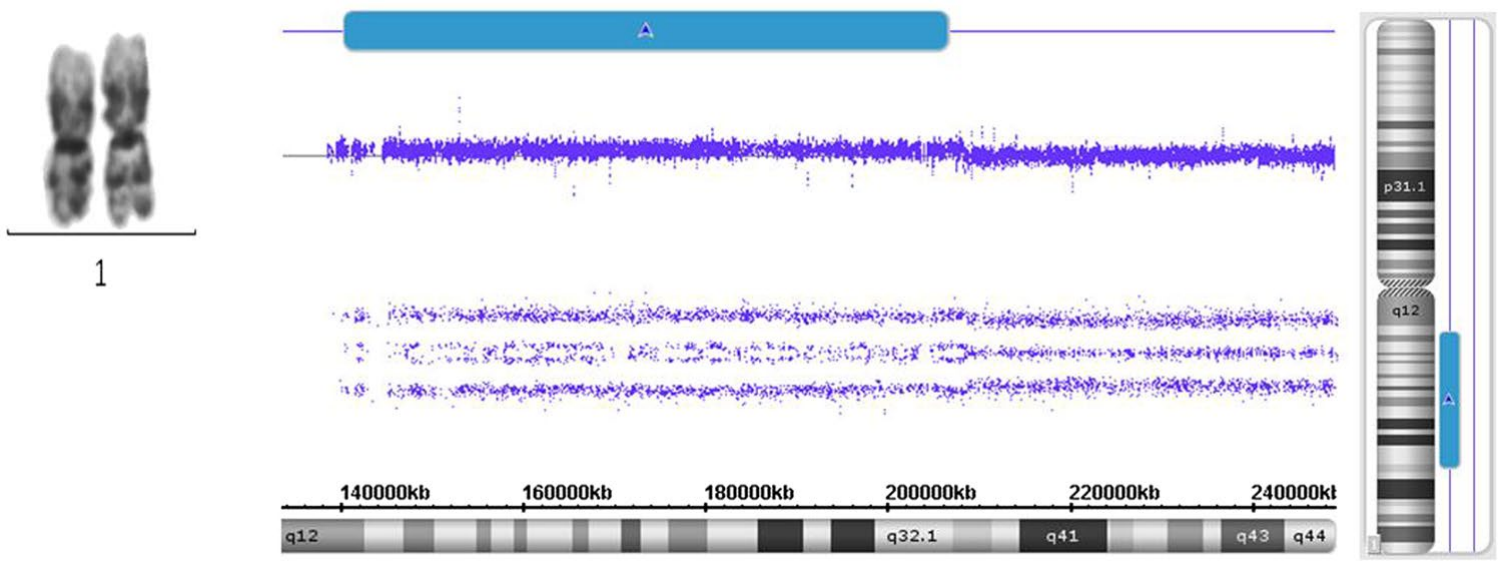
Comparison of chromosomal gain detected between MC and microarray. Although MC indicates normal chromosomal 1 in case 8#, Affymetrix Cytoscan 750 K Microarray still reveals cryptic gain(1q21.1-q32.2) with a large size.

**Figure 2:**
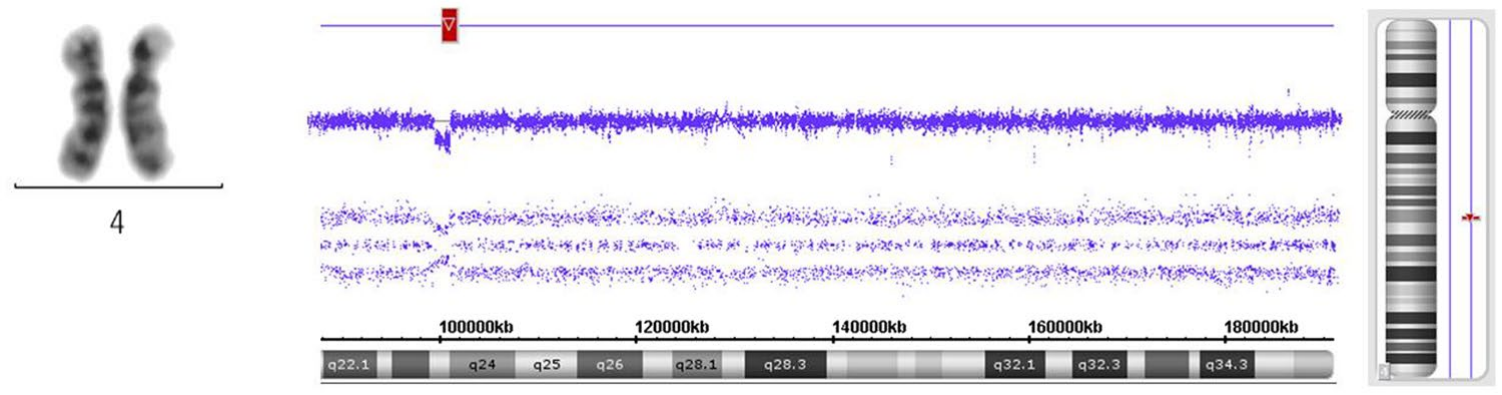
Comparison of chromosomal loss detected between MC and microarray. Although MC exhibits normal chromosomal 4 in case 10#, Affymetrix Cytoscan 750 K Microarray still discloses cryptic loss(4q24) with a small size.

High resolution genome-wide Affymetrix Cytoscan 750 K Microarray is able to detect CNV larger than 100 Kb^11^. As for case 1#, 3#, 4#, 5#, MC presented concordant results with microarray, yet microarray provided more precise chromosomal lesions in the samples. For instance, in case 1#, MC only revealed trisomy 8. By contrast, Cytoscan 750 K Microarray exhibited additional cryptic gain(3q27.1-qter) and loss(6q23.2-qter). Specific lesions can be seen in Table 1, Supplementary Figure S1 and Table S1. In case 2#, MC revealed discordant result: t(3;21)(q26;q22). Cytoscan 750 K Microarray didn’t demonstrate this translocation, but unraveled cryptic gain(Yq11.222-pter) and loss (Yq11.222-qter) (detail in Supplementary Figure S2.1,2 and Table S2). From case 6# to case 10#, MC presented normal karyotypes. By contrast, Cytoscan 750 K Microarray disclosed loss(20q11.23-q13.13) in case 6#, loss(13q13.1-q21.33) in case 7#, loss(14q11.2) and gain(1q21.1-q32.2) in case 8# (Fig. 1), loss(5q15-q22.3) in case 9#, loss(4q24, 7q11.21) in case 10# (Fig. 2 and Table 1). The concrete size, location, and copy number state of these chromosomal lesions are available in our supplementary file. Those cryptic aberrations disclosed by Cytoscan 750 K Microarray are important for characterizing the patients with a diagnosis of MDS.

**Table 1:** Comparison of CNV detected between Karyotypic analysis and Microarray.

Patient NO.	Gender	Age (y)	Diagnosis	MC	Microarray	
					gain	loss
1#	female	71	sAML	47,XX, +8[20]	3q27.1-qter +8	6q23.2-qter
2#	male	86	sAML	46,XY,t(3;21)(q26;q22) [19]/46,XY[1]	Yq11.222-pter	Yq11.222-qter
3#	male	69	RAEB-2	46,XY, −20, +mar[16] /46,XY[4]	20q13.2qter	20p11.1-pter; 20q11.21-q13.2
4 #	male	81	RCMD	46,X,-Y, +8[12]/46,XY[8]	+8	Yp11.31-q11.23
5#	female	60	RCMD	46,XX,del(20)(q11)[20]	1q21.2; 20p11.1	20q11.23-q13.32
6#	male	85	RAEB-1	46,XY		20q11.23-q13.13
7#	female	71	RAEB-1	46,XX		13q13.1-q21.33
8#	female	51	RA	46,XX	1q21.1-q32.2	14q11.2
9#	male	31	5q- syndrome	46,XY		5q15-q22.3
10#	male	78	RARS	46,XY		4q24; 7q11.21

### UPD

**Figure 3:**
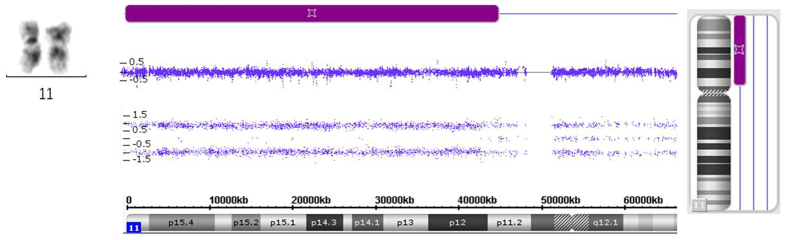
UPD disclosed by microarray. Although MC indicates normal chromosome 11, cryptic UPD(11p11.2-pter) in the chromosomal short arm of case 13# is still disclosed by Affymetrix Cytoscan 750 K Microarray.

UPD still remains indiscernible by classical cytogenetic techniques, because it doesn’t change chromosomal banding patterns^12^. In this cohort, UPD in 10 MDS patients have not been detected by MC but by Cytoscan 750 K Microarray. For instance, in case 13#, although MC exhibits normal chromosome 11, Cytoscan 750 K Microarray still revealed UPD(11p11.2-pter) that approximately covered the entire short arm of chromosome 11 (Fig. 3). Additional recurrent UPDs relevant to MDS have also been identified by microarray, such as UPD(4q12-qter) in case 11#, UPD(13q11-qter) in case 12#, UPD(6p21.31-pter) in case 14#, UPD(11q13.1-qter) in case 15#, UPD(15q11.2-qter) in case 19#, and UPD(22q12.1-qter) in case 20#. Detailed aberrations of UPD have been listed in Table 2 and our supplementary file.

**Table 2:** UPD exclusively identified by Cytoscan 750 K Microarray.

Patient NO.	Gender	Age (y)	Diagnosis	MC	Microarray (UPD)
11#	male	86	RCMD-RS	46,XY	4q12-qter; 6p22.2-p21.33
12#	female	9	RARS	46,XX	13q11-qter;Xq11.1-q13.1; Xq13.1-q21.1
13#	male	4	sAML	46,XY	11p11.2-pter; 2q11.1-q11.2
14#	male	34	RA	46,XY	6p21.31-pter
15#	female	76	RAEB-2	46,XX	11q13.1-qter
16#	female	51	RCMD	46,XX	3p21.31-p21.1;17q22-qter
17#	male	43	MDS-U	46,XY	3p21.31-p21.1;9p21.1-pter; 11p11.2-p11.12
18#	male	77	RARS	46,XY	11q12.3-q13.3; 14q24.1-qter
19#	male	75	RA	46,XY	11p11.2-p11.12;15q11.2-qter
20#	male	85	RA	46,XY	11p11.2-p11.12;22q12.1-qter

### Complex chromosomal lesions

Complex karyotypes in MDS often encompass three or more chromosomal abnormalities and are associated with unfavorable clinical outcomes^13^. Karyotypic analysis and Cytoscan 750 K Microarray have illustrated complex chromosomal lesions in 5 cases in point. Despite the fact that many large genomic aberrations were detected by metaphase cytogenetic assay, more hidden chromosomal loss and gain, especially UPD were still revealed by Cytoscan 750 K Microarray. In case 21#, MC indicates 40∼51, XY, ins(1)(p13p22p36), add(2)(q31), −5, −7, add(8)(p21), +9, add(9)(q34), −11, +mar1, +mar2, inc[cp6]. Comparatively, Cytoscan 750 K Microarray has revealed loss(1p22.3p21.2, 1p36.13p36.11, 5q14.3q21.3, 7q21.3q36.3, 12p13.31p12.1, 18q12.3qter), gain(8q11.1q24.3, 11p12q22.1, 13q11-q12.3), and UPD(7q22.1q31.32, 17p13.3p11.2) (Table 3, Supplementary Figure S21). As for case 22#, in spite of some “del” and “add” determined by karyotypic analysis, Cytoscan 750 K Microarray still disclosed a complex pattern of gains, losses and UPD in this case (Table 3, Supplementary Figure S22). Case 23#, 24#, and 25# have a series of complex chromosomal aberrations identified by Cytoscan 750 K Microarray that complement the results revealed by MC. By contrast, microarray analysis revealed cryptic UPD and a lot of smaller lesions, such as UPD(3p21.31-p21.1) and loss(3p14.1-q21.1, 5q13.3-q35.1, 9q21.11-q31.1) in case 25# (Table 3, Supplementary Figure S25). Generally, Cytoscan 750 K Microarray revealed complex rearrangements with multiple gains and losses. But MC indicated undefined materials exhibited as marker chromosomes (“+mar”) and chromosomal additions (“add”)^13^ in some cases. Detailed lesions of complex karyotypes have been listed in Table 3 and our Supplementary file.

**Table 3:** Complex chromosomal lesions detected by MC and Microarray.

Patient NO.	Gender	Age (y)	Diagnosis	Karyotypic analysis	Microarray		
					gain	loss	UPD
21#	male	80	sAML	40∼51,XY,ins(1)(p13p22p36),add(2) (q31),-5,-7,add(8)(p21), + 9,add(9)(q34), -11, + mar1, + mar2,inc[cp6]	8q11.1q24.3; 11p12q22.1; 13q11-q12.3	1p22.3p21.2; 1p36.13p36.11; 5q14.3q21.3; 7q21.3q36.3; 12p13.31p12.1; 18q12.3qter	7q22.1q31.32; 17p13.3p11.2
22#	male	71	sAML	41∼45,XY,del(2)(q33),-5,del(7)(p13),add(11)(q23), + 2∼3mar,inc[cp5]/46,XY[2]	15q22.2-q23; 21q11.2q22.11	3p11.1-pter; 5q14.2-qter; Entire 7; 12p13.2p12.2; 15q24.1q25.1; 21q22.11q22.13	20q11.21-q11.23
23#	male	28	sAML	42,X,add(Y)(p11),-5,-7,-11,-12,del(12)(p11),add(16)(q24),add(17)(q25),add(17)(p13),-22, + r[16]/46,XY[4]	11q22.3-qter; 16q23.1-qter	5q11.1-qter; Entire 7; 12p13.2-p11.23; 17p12-pter	
24#	male	84	sAML	54∼56,XY, + 1, + 2,der(4;12)(q10;q10), + 5,del(5)(q13q31) × 2,add(7)(q32), + 8, + 11,del(12)(p11), + 17,i(17)(q10) × 2, + add(18)(q23), + 21, + 22[cp18]/46,XY[2]	Entire 1,2, 6, 8,10,11,18; 5q14.2pter; 9p23p21.2; 21q11.2qter; 22q11.1qter	5q14.3qter; 12p13.31p11.21; 17q12 pter	9p21.2qter
25#	female	53	sAML	41∼44,X,-X,-5,add(14)(p11),-16,-18,?del(20)(q11),add(21)(p11), + r, + mar,1dmin[cp9]/46,XX[1]	5q12.3q13.2; 18q11.2-pter; 22q11.1-qter	3p14.1-q21.1; 5q13.3-q35.1; 9q21.11-q31.1; Entire 16; 17p13.3p13.1; 18q11.2-qter; 20q11.21-qter; Xp21.3p11.21; Xq13.1-qter	3p21.31-p21.1

### Validation of microarray results

**Figure 4:**
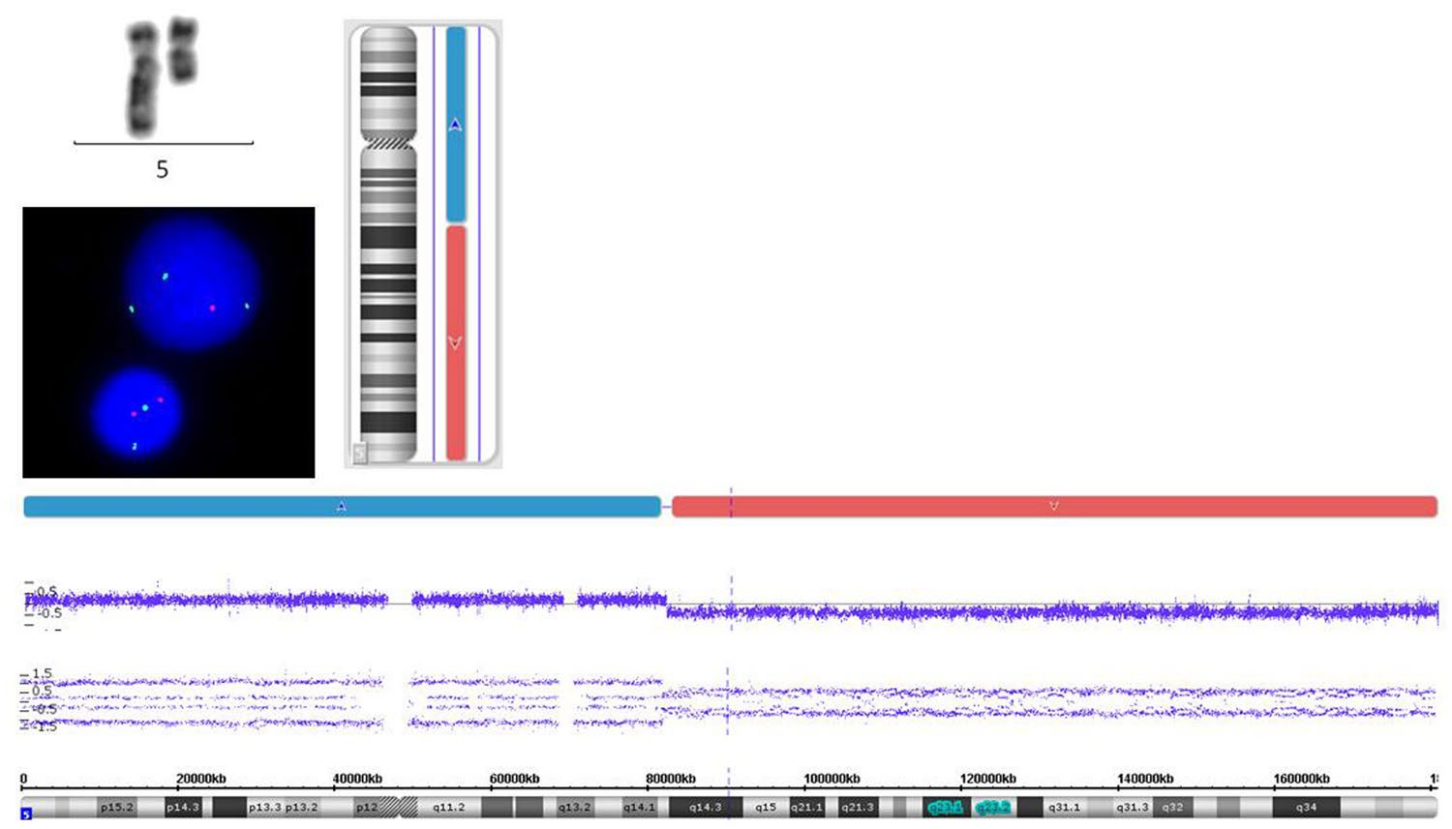
Validation of genomic aberrations in chromosomal 5. As for 24#, MC revealed del(5)(q13q31), while microarray disclosed gain(5q14.2pter) and loss(5q14.3qter). FISH has validated loss(5q33-34).

The resolution of conventional metaphase cytogenetic analysis is approximately 5 Mb^14^. In order to validate those lesions identified by microarray, we also initiated FISH to detect the chromosomal aberrations in some of those cases. For instance, in case 17#, UPD(3p21.31p21.1), UPD(9p21.1pter), and UPD(11p11.2p11.12) haven’t been detected by MC but by microarray. FISH also exhibit normal chromosome 5, 7, 8, 20 (Supplementary Figure S17, Table S17). In contrast, MC revealed +5 and del(5)(q13q31) in case 24#, while Cytoscan 750 K Microarray disclosed gain(5q14.2pter) and loss(5q14.3qter). FISH indicated loss of CSF1R signal, representing loss(5q33-34). So genomic loss in chromosomal 5 has been confirmed by FISH (Fig. 4). In addition, trisomy 8 in case 24# has also been validated by FISH (Supplementary Figure S24).

## Discussion

Chromosomal abnormalities are frequent in MDS and have many clinical implications. However, a great amount of patients don’t indicate cytogenetic abnormalities. MDS patients with the same chromosome lesions may have heterogeneous phenotypes, implying that some hidden genomic changes may exist among the patients^15^. In the present study, we utilized traditional MC and Affymetrix Cytoscan 750 K Microarray to identify latent chromosomal changes in a cohort of 25 patients. Our results indicated that high-resolution Affymetrix Cytoscan 750 K Microarray improves the identification of chromosomal aberrations by karyotypic analysis in MDS.

Gain and loss of gene copies may result in gene over-expression, absence of any functional transcript, or modest changes in gene expression^16^. We have unveiled chromosomal aberrations in regions defined as CNV of contiguous clones undetectable by classical karyotypic analysis. For instance, we identified cryptic gain (3q27.1-qter) and loss (6q23.2-qter) in case 1#. In this region, Loss(6q23.2-q23.3) has been reported to involve in sAML^17^. Furthermore, cryptic chromosomal aberrations detected by microarray have been useful for a comprehensive analysis of gene mutations in MDS. The copy number status of TET2 (on 4q24), IRF1 (5q31.1), NPM1 (5q35.1), LAMB4 (7q31.1), EZH2 (7q36.1), ETV6 (12p13.2), TP53 (17p13.1), NF1 (17q11.2), ASXL1 (20q11.21), RUNX1 (21q22.12), and STAG2 (Xq25) have been investigated in a large cohort of MDS patients^18^. In our cohort, we have identified a 1.48 Mb loss at 4q24 in case 10#. TET2 gene in this region is a tumor-suppressor gene^19^. The deletion or mutation of TET2 often predict inferior prognosis in patients with chronic myelomonocytic leukemia^20^. Although aberration in chromosome 5 has been considered as the most prevalent chromosomal lesion in MDS, some cryptic defects which affect additional key genes have not been clarified comprehensively. The major commonly deleted region (CDR) has been delineated at band 5q31.1. We have identified a 97.71 Mb loss(5q14.3qter) in case 24#. Deletion of 5q may engender haploinsufficiency of many critical genes, including ribosomal protein S14 (RPS14), casein kinase 1 α1 (CSNK1A1), adenomatous polyposis coli (APC), heat shock protein family A (HSP70) member 9 (HSPA9), early growth response 1 (EGR1), DEAD-box helicase 41 (DDX41), NPM1, TRAF-interacting protein with forkhead- associated domain B (TIFAB), Diaphanous-related formin 1 (DIAPH1), microRNA (miR)-145 and miR-146a^21^. Haploinsufficiency for the ribosomal gene RPS14 has been reported to impede erythroid differentiation in the 5q- syndrome^22^. Heterozygous deletion of CSNK1A1 may upregulate WNT signaling and stimulate stem cell expansion^23, 24^. Loss(5q14.3qter) in case 24# has been validated by FISH. We have also detected loss(7q11.21) in case 10# and monosomy 7 in case 22#, 23#. Deletion of 7q and monosomy 7 are also prevalent in MDS and often portend unfavorable outcome^25^. These chromosomal alterations can also cause haploinsufficiency of some key genes implicated in MDS. These genes comprise EZH2, CUX1 and MLL3^26–28^. MLL3 haploinsufficiency cooperates with RAS mutation and Trp53 to exacerbate leukemia^26^. In addition, we have found gain(20q13.2qter) in case 3#. MacKinnon *et al*. have investigated AML and MDS patients with 20q amplification. They identified a 250 kb common region which subsumed HCK, TM9SF4, PLAGL2, and POFUT1 gene. These patients often had a higher proportion of erythroblasts. The amplification of 20q portends the existence of oncogene^29^. Generally, these latent chromosomal aberrations contain a lot of key genes which are tightly associated with the pathogenesis of MDS. CNV identified by Affymetrix Cytoscan 750 K Microarray can contribute to the differential diagnosis of subtypes in MDS.

Recent investigations have indicated that UPD can be responsible for homozygosity of mutations of critical genes within chromosomal regions^30^. Reduction to homozygosity as a result of UPD was preliminarily considered to be a mechanism for the inactivation of tumor suppressor genes^30^. We have demonstrated UPD in 10 cases with normal karyotypes. For example, we detected UPD(13q11-qter) in case 12#. FLT3 gene in 13q12 encodes class III receptor tyrosine kinase that regulates hematopoiesis^31^. FLT3-ITD internal tandem duplications have been observed during disease progression and confers an unfavorable prognosis^32^. We also identified UPD(11p11.2-pter) in case 13#. WT1 gene mutation has been reported in UPD 11p which is related to the pathogenesis of AML^33^. Furthermore, we have also found UPD (11q13.1-qter) in case 15#. The c-CBL gene is located in 11q23.3^7^. Clonal selection of UPD 11q and CBL gene mutation often reflected the progression of MDS to AML^34^. It is noteworthy that in RCMD case 16#, the UPD of region 17q22-qter harbored the ETV4 gene, which encodes an ETS transcription factor indispensable for hematopoiesis^35^. Additionally, we have found UPD(9p21.1-pter) in case 17#. UPD 9p is tightly associated with a homozygous activating JAK2(V617F) gene mutation, implying serious prognosis^7^. Consequently, nonrandom segmental UPDs identified in this cohort may contribute to the investigation of the pathogenesis of MDS underlying large deletions.

MDS with complex chromosomal aberrations often herald short survival and an increased risk of evolution to AML^36^. Complex karyotypes with multiple chromosomal changes are found in about 20% of newly diagnosed MDS patients and are relevant to a poor prognosis^3^. We exhibited 5 cases with complex chromosomal lesions, which have been listed in our supplementary file. A combination of traditional karyotypic analysis with Affymetrix Cytoscan 750 K Microarray may well provide a more comprehensive detection of complex chromosomal aberrations in MDS.

Given the recent discovery of many recurrent gene mutations in MDS, it’s still urgent to validate prior mutational correlative data. The temporal order of mutation acquisition has reflected the importance of subclonal genetic events in MDS. For instance, mutations impacting RNA splicing and DNA methylation occur early in disease progression, while kinase activating mutations (such as KIT and NRAS) occur even later in disease progression^37^. Early detection of subclonal mutations may reflect significant prognostic variables in MDS^38^. In our cohort, we have found many discrepancies between MC and Cytoscan 750 K Microarray. Microarray analysis has a variable ability to detect mosaicism that FISH and karyotyping may not accurately detect the level of mosaicism^13^. The most likely reason for these discrepancies is probably that some aberrations are subclonal.

On the other hand, one criticism of microarray for detecting chromosomal aberrations in MDS is the possibility of “false positive” results or findings of unclear clinical significance. A proportion of alterations identified in the patients may reflect normal age-related chromosomal changes. For instance, we have found loss(Yq11.222-qter) in case 2#. Loss of the Y-chromosome (LOY) is described as both a normal age-related event and a marker of a neoplastic clone in hematologic diseases^39^. Paired normal DNA from the same MDS patient may reduce the number of false positives generated by microarray^40^.

Additionally, some patients with clonal cytopenia of undetermined significance (CCUS) that do not meet the criteria for MDS may also benefit from SNP-A. A recent study has applied combined comparative genomic hybridization and SNP-A to detect cryptic chromosomal lesions in both MDS and cytopenias of undetermined significance. Based on the combined array findings, 42% of patients with indeterminate morphologic findings were categorized as CCUS. Cryptic array findings among those patients comprised large-scale UPD (up to 118 Mb) and genomic deletion of loci implicated in MDS pathogenesis (eg, TET2 (4q22) and NUP98 (11p15)). The latent chromosomal lesions revealed by SNP-A helped to indicate clonal hematopoiesis and prompted classification as CCUS^41^. Hence microarray analysis significantly improves the detection rate of clinically significant findings.

In conclusion, Affymetrix Cytoscan 750 K Microarray have identified many cryptic chromosomal abnormalities relevant to MDS, which may interpret the clinical variability and enhance our understanding of the pathogenesis of MDS.

## Material and Methods

### Patients and Specimen

The cohort of this study comprises patients whose bone marrow aspirates were recruited in Kingstar Global company for pathologic diagnosis of MDS from December 2014 to July 2015. All specimens were acquired with patients’ approval, under the protocols permitted by Institutional Ethics Committee of Wuhan university, in comply with Helsinki Declaration. Informed consent was signed for each patient. And any publication of identifying information was also approved by the participants.

### Cytogenetic analysis

Traditional G-banding Karyotypic analysis was initiated on bone marrow aspirates by trypsin and Giemsa dye. Short-term cell cultures were carried out in medium supplemented with GM-CSF or conditioned medium III. Then the cells were harvested and metaphase preparations were performed according to standard procedures. Karyotypes were depicted in the light of International System for Human Cytogenetic Nomenclature 2016^42^. At least 20 metaphases per sample should be analyzed whenever possible.

**FISH** Fluorescence *in situ* hybridization (FISH) was performed according to the manufacturer’s protocols, in order to validate chromosomal aberrations detected by Affymetrix Cytoscan 750 K Microarray. A total of 400 interphase nuclei were evaluated by two independent pathologists under fluorescent microscope. The locus-specific probes were displayed in Table 4.

**Table 4:** The probes and targets of FISH.

Probe	Target
D5S23,D5S721/CSF1R	5p15.2/5q33-34
D7Z1/D7S486	7p11.1-q11.1/7q31
D8Z2	8p11.1-q11.1
D20S108	20q12

### DNA preparation

DNA was extracted from bone marrow of individual patients using the QIAamp DNA Blood Mini Kit according to the manufacturer’s instructions. The concentration and quality of DNA samples were evaluated by Nanodrop 2000 spectrophotometer (Thermo Scientific). DNA integrity was assessed by 1% agarose gel electrophoresis. The quality controls (QC) of Affymetrix CytoScan 750 K microarray required that DNA concentration should be no less than 50 ng/µL, OD260/280 is about 1.9, OD260/230 is about 2.0.

### Cytoscan 750 K Microarray Assay

**Figure 5:**
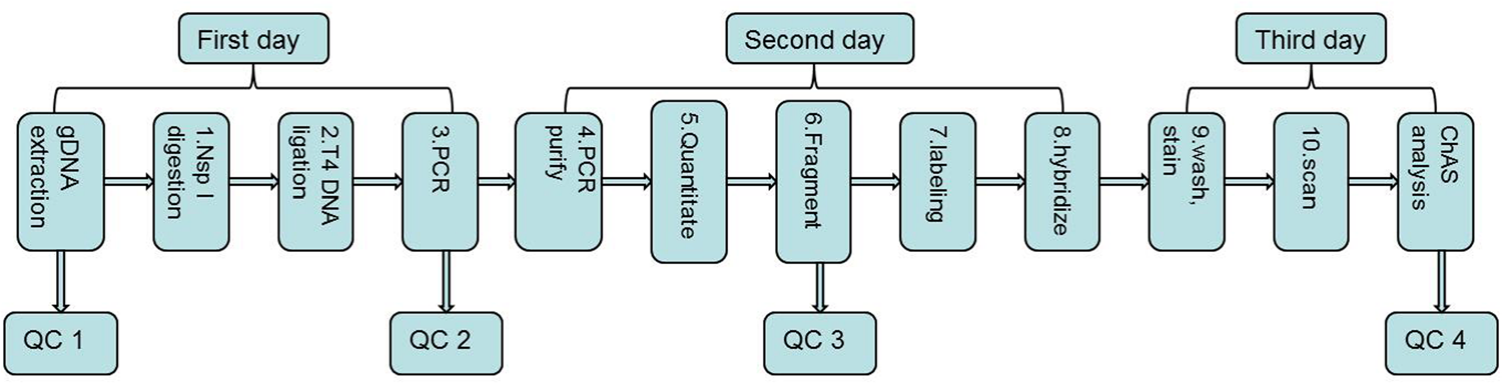
The Workflow of Affymetrix Cytoscan 750 K Microarray. **QC1** defines DNA concentration ≥ 50ng/µL, OD260/280≈1.9, OD260/230 ≈ 2.0. **QC2** defines PCR products on 1% gel electrophoresis should be 150 bp-2000 bp, Purified PCR products ≥ 300 ng/µL, OD260/280 ≈ 1.9, OD260/230≈2.0. **QC3** defines fragmentation products on 1% gel electrophoresis should be 25 bp-125 bp. **QC4** defines: SNPQC ≥ 15.0; MAPD ≤ 0.25; Waviness SD ≤ 0.12.

Affymetrix Cytoscan 750 K Microarray provides a genome-wide coverage with focus on cytogenetic relevant regions, including 550,000 markers for detecting copy number variation and 200,000 high performing SNP probes with genotype accuracy >99%. All probes are empirically selected for exceptional performance. The Affymetrix® CytoScan™ Assay protocol is optimized for processing 8 to 24 samples at a time to obtain whole genome copy number and SNP information. The Workflow of CytoScanTM Assay can be briefed as follows (Fig. 5):

1. Digestion of gDNA with Nsp I restriction endonuclease.
2. Ligation with Adaptor and T4 DNA Ligase.
3. Polymerase chain reaction (PCR) to amplify Ligated Samples and PCR Product Check.
4. PCR Product Purification with magnetic beads.
5. Quantitation of purified samples.
6. Fragmentation of Purified PCR Products and QC Gel Analysis.
7. Labeling the Fragmented DNA with TdT enzyme.
8. Hybridization with CytoScan 750 K Microarray at 50 °C oven for 16 to 18 hours.
9. Wash and Stain the genechips on Fluidics Station.
10. Scan the arrays in optic GeneChip Scanner 3000.

All cases have followed the protocols and QC guidelines provided by the manufacturer.

### Data analysis

The data of Cytoscan 750 K Microarray were analyzed using Chromosome Analysis Suite Version 2.0 (Affymetrix). The QC thresholds were: SNPQC ≥ 15.0; MAPD ≤ 0.25; Waviness SD ≤ 0.12. These QC metrics can evaluate the overall quality of SNP array data. Median Absolute Pairwise Difference (MAPD) represents the typical distance between marker pairs with respect to log2 ratios. SNPQC measures the degree of separation between genotype clusters aggregated across multiple markers. Waviness-SD gauges the differences between probe sets. The microarray data were interpreted according to the annotations of genome version GRCh37 (hg19). Only the samples which complied with QC criteria and identified CNV with over 100 Kb and at least 10 aberrant probes were chosen for further analysis. Identified CNVs were contrasted with the Database of Genomic Variants (http://dgv.tcag.ca/dgv/app/home) to exclude the polymorphic variations in healthy population. As for UPD, we used an algorithm that regards both location and size of >5 Mb aberrations in order to preclude nonclonal regions. To reckon the size of the affected genome in each patient, we recognized the total size of alterations in chromosomes, including CNV and UPD.

## Electronic supplementary material

MDS supplementary file
