# Supplementary material for: Identify latent chromosomal aberrations relevant to myelodysplastic syndromes
Source: Sci Rep. 2017 Sep 4;7:10354. doi: 10.1038/s41598-017-10551-3 (PMC5583229; doi:10.1038/s41598-017-10551-3)
Supplement: Supplementary file 1 — MDS supplementary file [file 41598_2017_10551_MOESM1_ESM.pdf]

Identify latent chromosomal aberrations relevant to myelodysplastic syndromes

Qibin Song[1*], Yuxin Chu[1], Yi Yao[1], Min Peng[1], Weihong Yang [2], Xiaoqing Li [2], Shiang Huang [2]

Case 1#，female, 71 years old, sAML

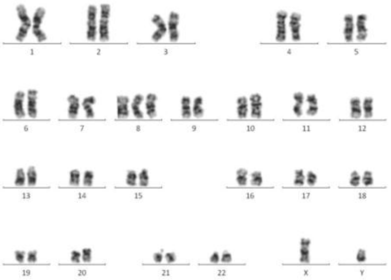
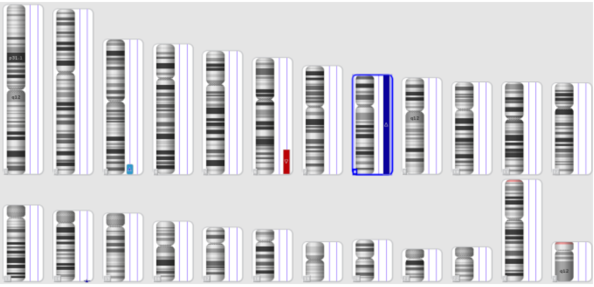

Figure S1.1: Abnormal Metaphase Cytogenetics indicates 47,XX,+8[20]

Figure S1.2:SNP-A results indicate Gain(3q27.1-qter), Loss(6q23.2-qter), Gain(8)

Table S1: Chromosomal lesions detected by SNP-A in Case 1#.

Type	Microarray Nomenclature	Size(Kbp)	CN state
GainMosaic	arr[hg19] 3q27.1q29(183,344,691-197,851,444)x2-3	14506	2.8
Loss	arr[hg19] 6q23.2q27(135,160,366-170,914,297)x1	35754	1
Gain	arr[hg19] 8p23.3q24.3(158,048-146,295,771)x3	146138	3

Case 2#，male, 86 years old, sAML

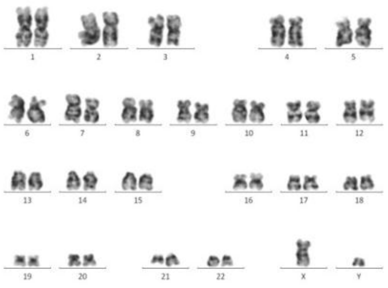
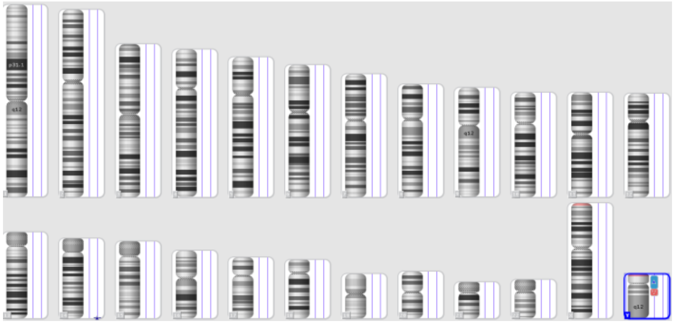

Figure S2.1: Abnormal Metaphase Cytogenetics indicates 46,XY,t(3;21)(q26;q22)[19]/46,XY[1]

Figure S2.2: SNP-A results indicate GainMosaic(Yq11.222-pter), LossMosaic(Yq11.222-qter), and Gain(14q32.33).

Table S2: Chromosomal lesions detected by SNP-A in Case 2#.

Type	Microarray Nomenclature	Size(Kbp)	CN state
Gain	arr[hg19] 14q32.33(106,251,069-106,769,864)x3	519	3
GainMosaic	arr[hg19] Yp11.31q11.222(2,650,424-19,972,006)x1-2	17321	1.5
LossMosaic	arr[hg19] Yq11.222q11.23(19,975,331-28,799,654)x0-1	8824	0.5

Case 3#，male，69 years old, RAEB-2

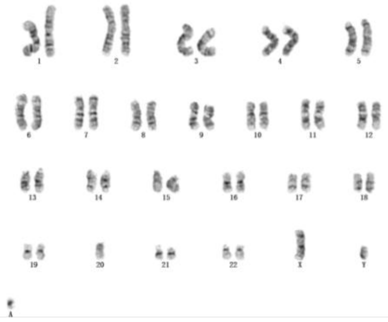

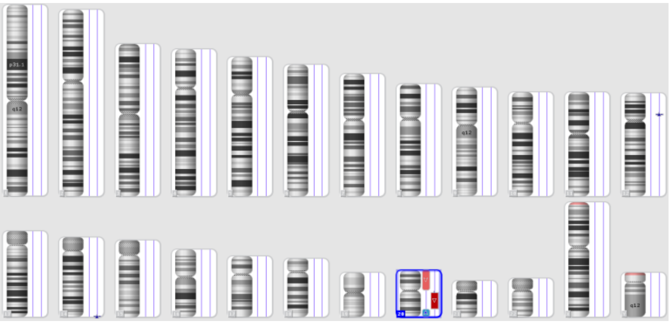

Figure S3.1: Abnormal Metaphase Cytogenetics indicates 46,XY,-20,+mar[16]/46,XY[4]

Figure S3.2: SNP-A results indicate LossMosaic(20p13p11.1), Loss(20q11.21q13.2), GainMosaic(20q13.2qter).

Table S3: Chromosomal lesions detected by SNP-A in Case 3#.

**Type**	**Microarray Nomenclature**	**Size(Kbp)**	**CN state**
LossMosaic	arr[hg19] 20p13p11.1(61,661-26,305,478)x1-2	26244	1.2
Loss	arr[hg19] 20q11.21q13.2(30,947,118-52,423,569)x1	21476	1
GainMosaic	arr[hg19] 20q13.2q13.33(53,178,091-62,913,645)x2-3	9735	2.4

Case 4#, male, 81years old, RCMD

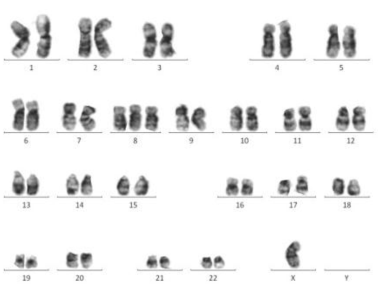

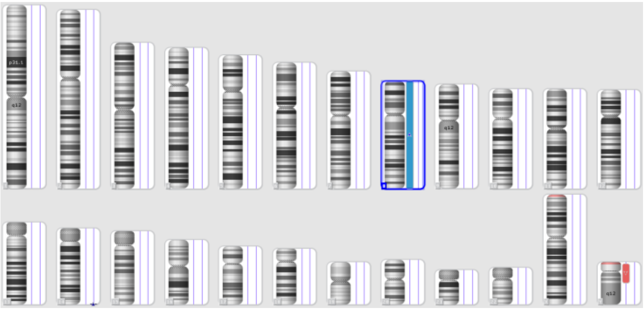

Figure S4.1: Abnormal Metaphase Cytogenetics indicates 46,X,-Y,+8[12]/46,XY[8].

Figure S4.2: SNP-A results indicate trisomy 8, Loss(Yp11.31q11.23).

Table S4: Chromosomal lesions detected by SNP-A in Case 4#.

**Type**	**Microarray Nomenclature**	**Size(Kbp)**	**CN state**
GainMosaic	arr[hg19] 8p23.3q24.3(158,048-146,295,771)x2-3	146137	2.3
LossMosaic	arr[hg19] Yp11.31q11.23(2,650,424-28,799,654)x0-1	26149	0.25

Case 5#，female，60years old, RCMD

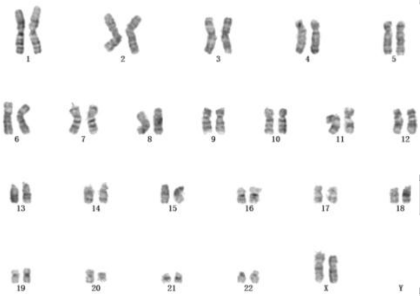

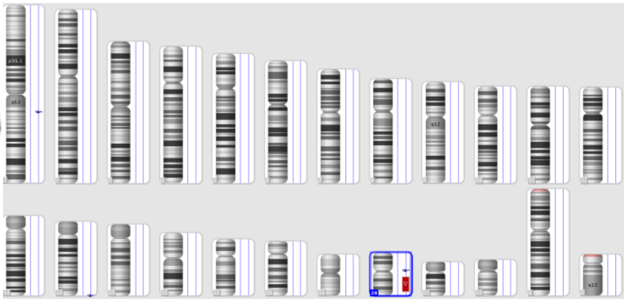

Figure S5.1: Abnormal Metaphase Cytogenetics indicates 46,XX,del(20)(q11)[20].

Figure S5.2: SNP-A results indicate Gain(1q21.2), Gain(20p11.1), Loss(20q11.23q13.32).

Table S5: Chromosomal lesions detected by SNP-A in Case 5#.

Type	Microarray Nomenclature	Size(Kbp)	CN state
Gain	arr[hg19] 1q21.2(148,545,141-149,733,322)x3	1188	3
Gain	arr[hg19] 20p11.1(25,663,183-26,076,490)x3	413	3
Loss	arr[hg19] 20q11.23q13.32(35,603,579-56,680,689)x1	21077	1

Case 6#，male，85years old, RAEB-1

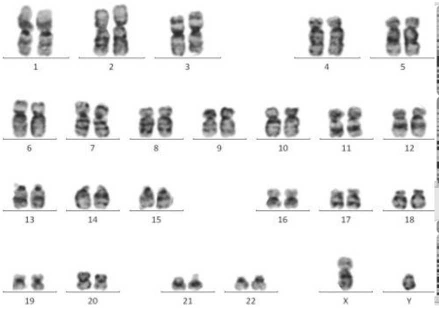

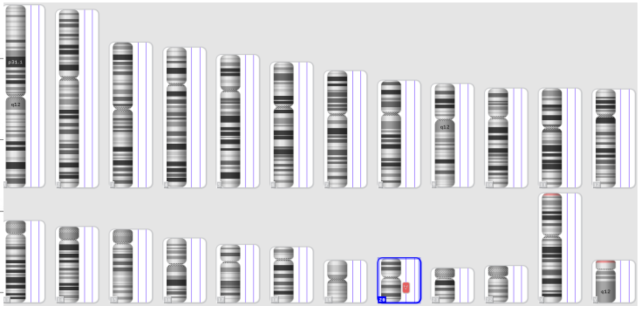

Figure S6.1: Normal Metaphase Cytogenetics indicates 46,XY[20].

Figure S6.2: SNP-A results indicate cryptic Loss(20q11.23q13.33).

Table S6: Chromosomal lesions detected by SNP-A in Case 6#.

Type	Microarray Nomenclature	Size(Kbp)	CN state
LossMosaic	arr[hg19] 20q11.23q13.13(34,585,051-48,970,638)x1-2	14,386	1.85

Case 7#，female，71years old，RAEB-1

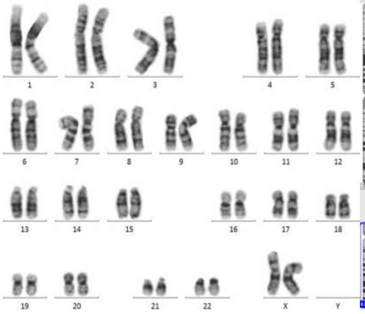

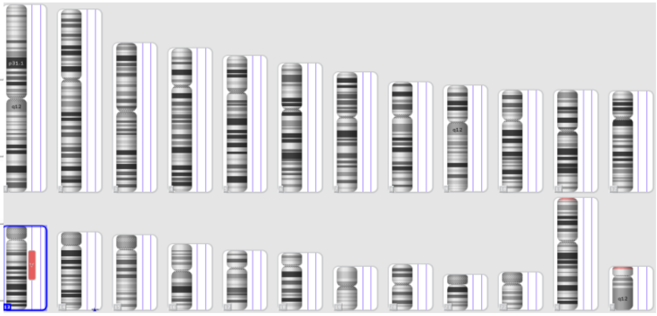

Figure S7.1: Normal Metaphase Cytogenetics indicates 46,XX [20].

Figure S7.2: SNP-A results indicate cryptic Loss(13q13.1-q21.33).

Table S7: Chromosomal lesions detected by SNP-A in Case 7#.

Type	Microarray Nomenclature	Size(Kbp)	CN state
LossMosaic	arr[hg19] 13q13.1q21.33(33,520,119-73,254,426)x1-2	39,734	1.75

Case 8#，female，51 years old，RA

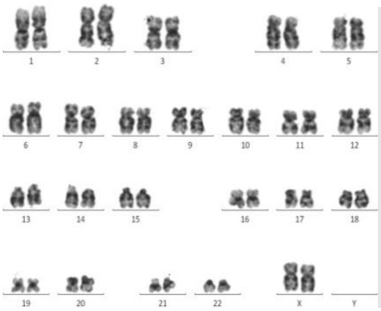

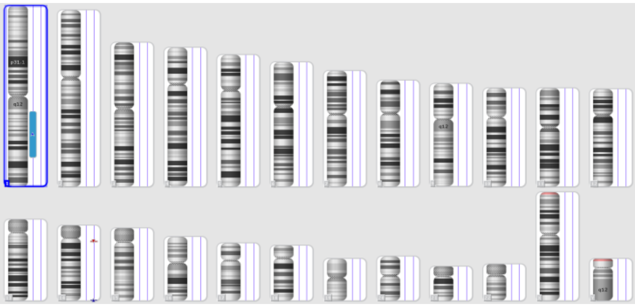

Figure S8.1: Normal Metaphase Cytogenetics indicates 46,XX [20].

Figure S8.2: SNP-A results indicate cryptic Gain(1q21.1-q32.2) and Loss(14q11.2).

Table S8: Chromosomal lesions detected by SNP-A in Case 8#.

Type	Microarray Nomenclature	Size(Kbp)	CN state
GainMosaic	arr[hg19] 1q21.1q32.2(145,888,925-208,990,187)x2-3	63,101	2.43
Loss	arr[hg19] 14q11.2(22,448,722-22,970,327) x1	522	1

Case 9#, male，31 years old，5q- syndrome.

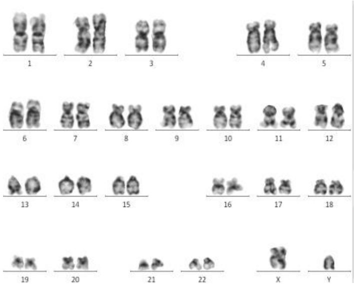

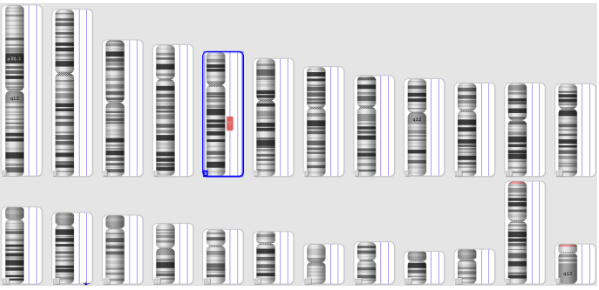

Figure S9.1: Normal Metaphase Cytogenetics indicates 46,X**Y** [20].

Figure S9.2: SNP-A results indicate cryptic Loss(5q15-q22.3).

Table S9: Chromosomal lesions detected by SNP-A in Case 9#.

**Type**	**Microarray Nomenclature**	**Size(Kbp)**	**CN state**
LossMosaic	arr[hg19] 5q15q22.3(94,204,970-114,496,815) x1-2	20,292	1.8

Case 10#，male，78 years old**,** RARS

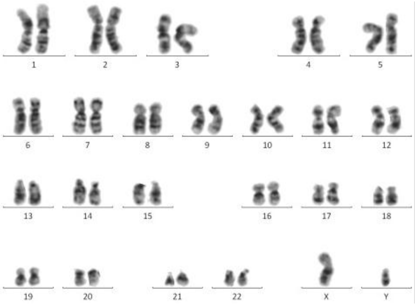

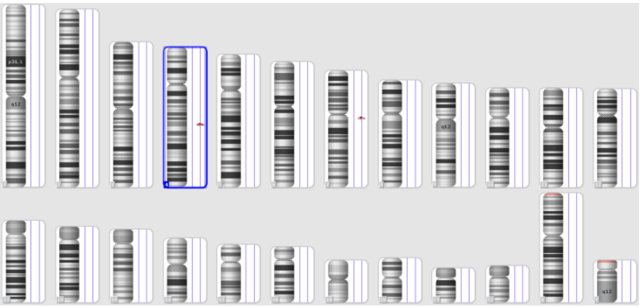

Figure S10.1: Normal Metaphase Cytogenetics indicates 46,X**Y** [20].

Figure S10.2: SNP-A results indicate cryptic Loss(4q24).

Table S10: Chromosomal lesions detected by SNP-A in Case 10#.

**Type**	**Microarray Nomenclature**	**Size(Kbp)**	**CN state**
Loss	arr[hg19] 4q24(105,030,235-106,514,099)x1	1484	1
Loss	arr[hg19] 7q11.21(64,651,296-65,148,399)x1	497	1

Case 11#，male，86 years old**,** RCMD-RS

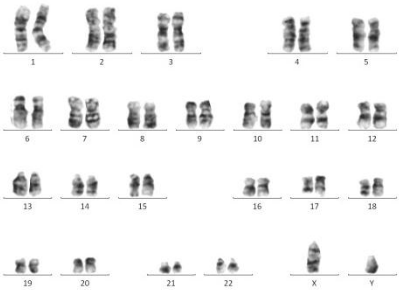 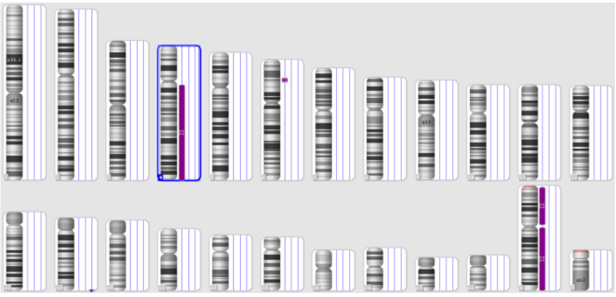

Figure S11.1: Normal Metaphase Cytogenetics indicates 46,X**Y** [20].

Figure S11.2: SNP-A results indicate cryptic UPD(4q12-qter) and UPD(6p22.2p21.33).

Table S11: Chromosomal lesions detected by SNP-A in Case 11#.

**Type**	**Microarray Nomenclature**	**Size(Kbp)**	**CN state**
UPD	arr[hg19] 4q12qter(55,497,445-190,921,709) hmz	135,424	2
UPD	arr[hg19] 6p22.2p21.33(25,944,802-31,916,916) hmz	5,972	2

Case 12#，female，9 years old, RARS

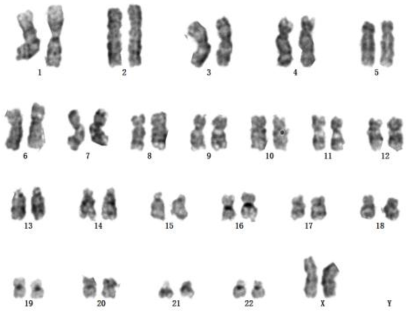 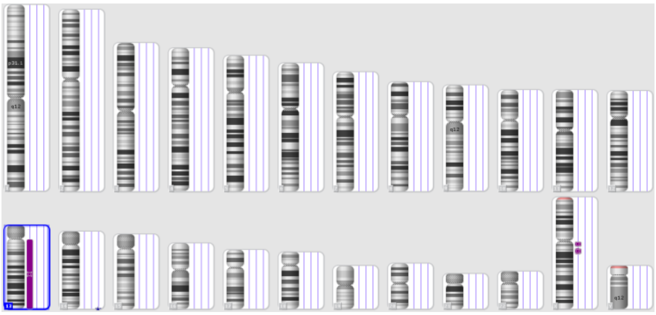

Figure S12.1: Normal Metaphase Cytogenetics indicates 46,X**X** [20].

Figure S12.2: SNP-A results indicate cryptic UPD(13q11qter), UPD(Xq11.1q13.1), and UPD(Xq13.1q21.1).

Table S12: Chromosomal lesions detected by SNP-A in Case 12#.

**Type**	**Microarray Nomenclature**	**Size(Kbp)**	**CN state**
UPD	arr[hg19] 13q11qter(19,450,956-115,095,705) hmz	95,645	2
UPD	arr[hg19] Xq11.1q13.1(62,036,670-68,187,282) hmz	6,151	2
UPD	arr[hg19] Xq13.1q21.1(71,380,957-78,955,504) hmz	7,575	2

Case 13#，male，4 years old，sAML

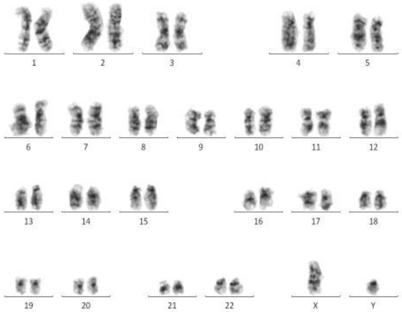

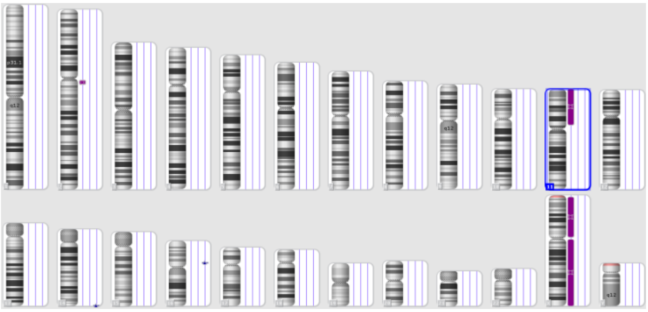

Figure S13.1: Normal Metaphase Cytogenetics indicates 46,XY [20].

Figure S13.2: SNP-A results indicate cryptic UPD(2q11.1q11.2), and UPD(11p11.2pter).

Table S13: Chromosomal lesions detected bySNP-A in Case 13#.

**Type**	**Microarray Nomenclature**	**Size(Kbp)**	**CN state**
UPD	arr[hg19] 2q11.1q11.2 (95,550,957-100,690,354) hmz	5,139	2
UPD	arr[hg19] 11p11.2pter (230,750-47,934,723) hmz	47,704	2

Case 14#，male，34 years old，RA

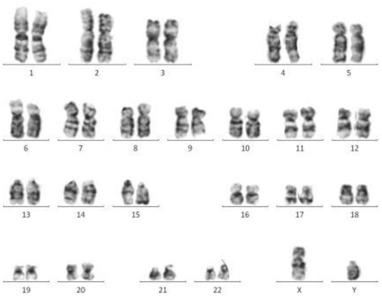

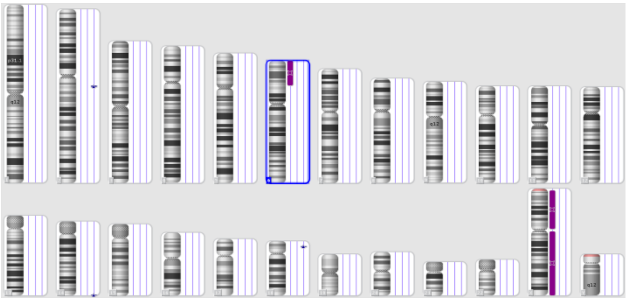

Figure S14.1: Normal Metaphase Cytogenetics indicates 46,XY [20].

Figure S14.2: SNP-A results indicate cryptic UPD(6p21.31pter).

Table S14: Chromosomal lesions detected bySNP-A in Case 14#.

**Type**	**Microarray Nomenclature**	**Size(Kbp)**	**CN state**
UPD	arr[hg19] 6p21.31pter (203,877-35,213,540) hmz	35,010	2

Case 15#，female，76 years old，RAEB-2

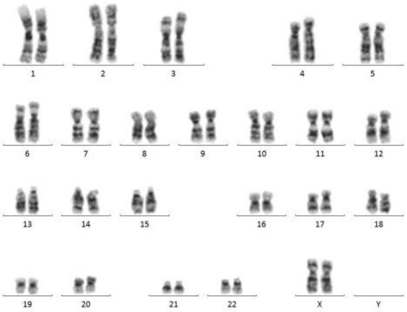

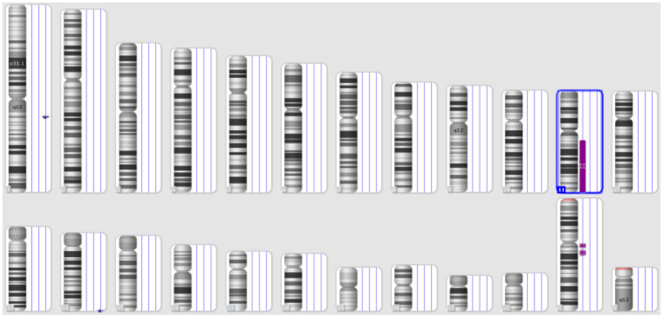

Figure S15.1: Normal Metaphase Cytogenetics indicates 46,XX [20].

Figure S15.2: SNP-A results indicate cryptic UPD(11q13.1qter).

Table S15: Chromosomal lesions detected by SNP-A in Case 15#.

Type	Microarray Nomenclature	Size(Kbp)	CN state
UPD	arr[hg19]<br>11q13.1qter (65,598,440-134,930,689) hmz	69,332	2

Case 16#，female，51 years old，RCMD

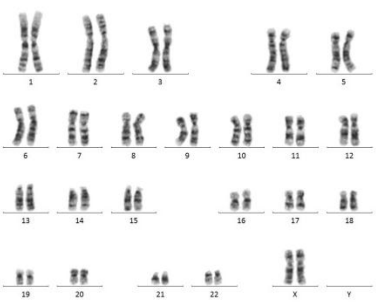

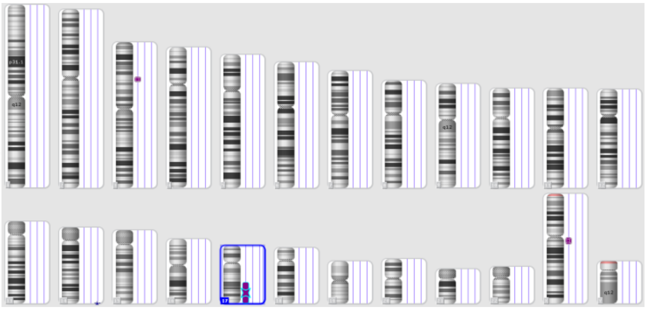

Figure S16.1: Normal Metaphase Cytogenetics indicates 46,XX [20].

Figure S16.2: SNP-A results indicate cryptic UPD(3p21.31p21.1) and UPD(17q22qter).

Table S16: Chromosomal lesions detected by SNP-A in Case 16#.

Type	Microarray Nomenclature	Size(Kbp)	CN state
UPD	arr[hg19]<br>3p21.31p21.1(46,895,299-53,285,424) hmz	6,390	2
UPD	arr[hg19]<br>17q22qter (51,863,665-81,041,760) hmz	29,178	2

Case 17#，male，43 years old, MDS-U

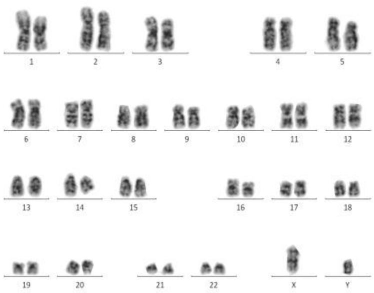

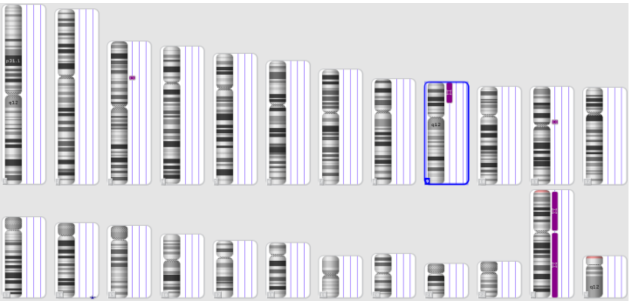

Figure S17.1: Normal Metaphase Cytogenetics indicates 46,XY [20].

Figure S17.2: SNP-A results indicate cryptic UPD(3p21.31p21.1), UPD(9p21.1pter), and UPD(11p11.2p11.12).

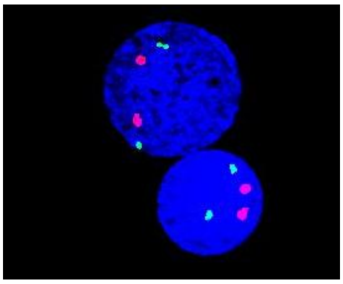

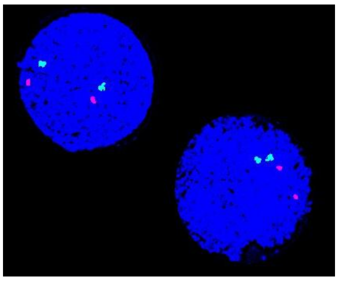

D5S23, D5S721/CSF1R D7Z1/D7S486

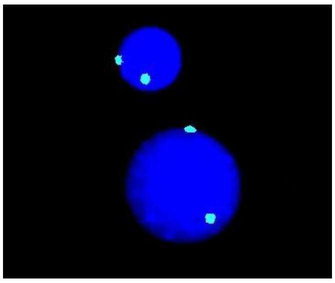

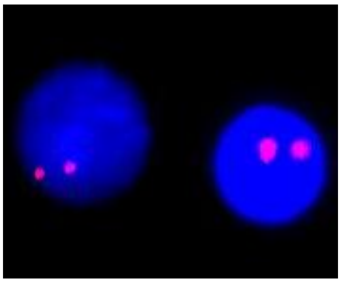

D8Z2 D20S108

Figure S17.3: FISH indicates normal chromosome 5,7,8,20.

nuc ish(D5S23,D5S721×2),(CSF1R×2)[400]; nuc ish(D7Z1×2),(D7S486×2)[400]

nuc ish(D8Z2×2)[400]; nuc ish(D20S108×2)[400]

Table S17: Chromosomal lesions detected by SNP-A in Case 17#.

**Type**	**Microarray Nomenclature**	**Size(Kbp)**	**CN state**
UPD	arr[hg19] 3p21.31p21.1(47,388,439-52,595,417) hmz	5,207	2
UPD	arr[hg19] 9p21.1pter (216,123-28,254,742) hmz	28,039	2
UPD	arr[hg19] 11p11.2p11.12 (45,662,054-51,550,787) hmz	5,889	2

Case 18#，male，77 years old，RARS

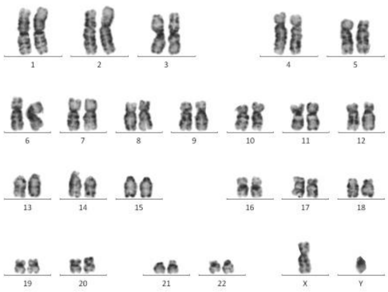
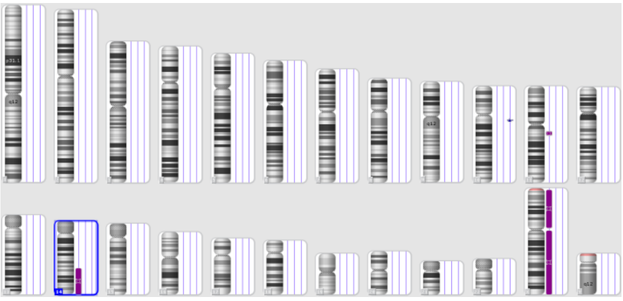

Figure S18.1: Normal Metaphase Cytogenetics indicates 46,XY [20].

Figure S18.2: SNP-A results indicate cryptic UPD(11q12.3q13.3) and UPD(14q24.1-qter).

Table S18: Chromosomal lesions detected by SNP-A in Case 18#.

Type	Microarray Nomenclature	Size(Kbp)	CN state
UPD	arr[hg19] 11q12.3q13.3(63,231,375-68,536,839) hmz	5,305	2
UPD	arr[hg19] 14q24.1-qter (69,088,578-107,279,475) hmz	38,190	2

Case 19#，male，75 years old，RA

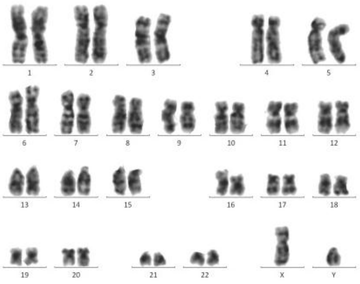
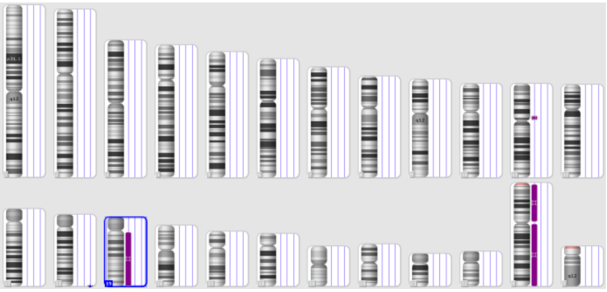

Figure S19.1: Normal Metaphase Cytogenetics indicates 46,XY [20].

Figure S19.2: SNP-A results indicate cryptic UPD(11p11.2p11.12) and UPD(15q11.2qter).

Table S19: Chromosomal lesions detected by SNP-A in Case 19#.

Type	Microarray Nomenclature	Size(Kbp)	CN state
UPD	arr[hg19] 11p11.2p11.12(46,240,816-51,550,787) hmz	5,310	2
UPD	arr[hg19] 15q11.2qter (22,817,870-102,397,317) hmz	79,579	2

Case 20#，male，85 years old，RA

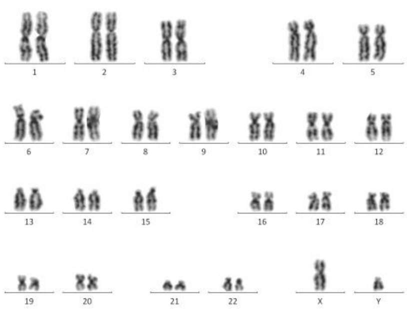

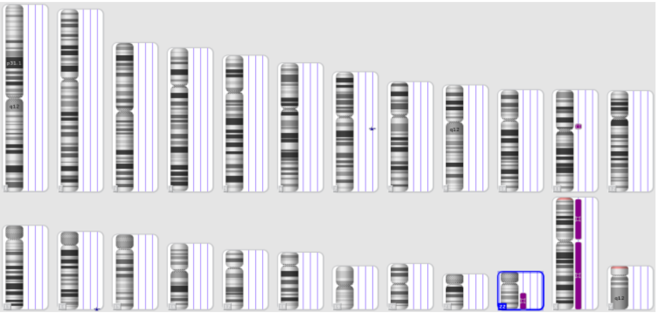

Figure S20.1: Normal Metaphase Cytogenetics indicates 46,XY [20].

Figure S20.2: SNP-A results indicate cryptic UPD(11p11.2p11.12) and UPD(22q12.1qter).

Table S20: Chromosomal lesions detected by SNP-A in Case 20#.

**Type**	**Microarray Nomenclature**	**Size(Kbp)**	**CN state**
UPD	arr[hg19] 11p11.2p11.12(45,267,714-51,550,787) hmz	6,283	2
UPD	arr[hg19] 22q12.1qter (29,287,729-51,157,531) hmz	21,870	2

Case 21#，male，80 years old，sAML

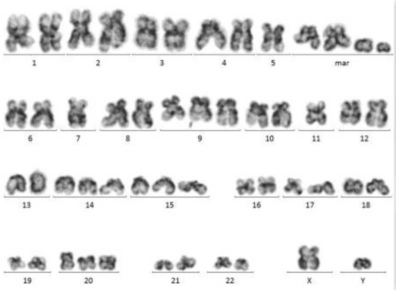

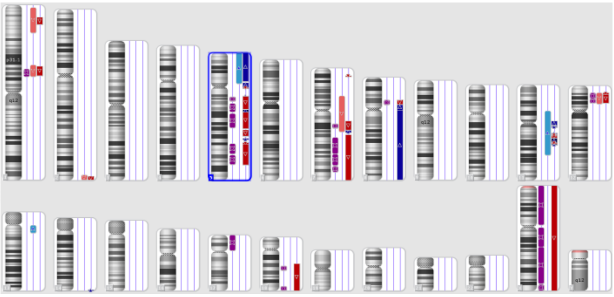

Figure S21.1: Metaphase Cytogenetics indicates 40～51,XY,ins(1)(p13p22p36),add(2)(q31),-5,-7,add(8)(p21),+9,add(9)(q34),?-11,+mar1,+mar2,inc[cp6]

Figure S21.2: SNP-A results indicate complex chromosomal aberrations (Table S21).

Table S21: Chromosomal lesions detected by SNP-A in Case 21#.

**Type**	**Microarray Nomenclature**	**Size(Kbp)**	**CN state**
Loss	arr[hg19] 1p22.3p21.2 (87,121,953-100,302,534)x1	13180	1
Loss	arr[hg19] 1p36.13p36.11 (17,341,540-26,792,047)x1	9450	1
Loss	arr[hg19] 5q14.3q21.3 (84,394,241-107,004,472)x1	22610	1
Loss	arr[hg19]	64295	1

	7q21.3q36.3 (94,824,955-159,119,707)x1		
UPD	arr[hg19]<br>7q22.1q31.32 (98,809,403-121,480,975) hmz	22671	2
Gain	arr[hg19]<br>8q11.1q24.3 (47,669,895-146,295,771)x3	98626	3
GainMosaic	arr[hg19]<br>11p12q22.1 (36,846,374-99,250,161)x2-3	62404	2.3
LossMosaic	arr[hg19]<br>12p13.31p12.1 (10,091,075-25,410,539)x1-2	15319	1.4
GainMosaic	arr[hg19]<br>13q11q12.3 (19,436,286-29,890,054)x2-3	10454	2.2
UPD	arr[hg19]<br>17p13.3p11.2 (18,900-22,170,994) hmz	22152	2
Loss	arr[hg19]<br>18q12.3q23 (38,932,938-78,013,728)x1	39081	1

Case 22#，male，71 years old，sAML

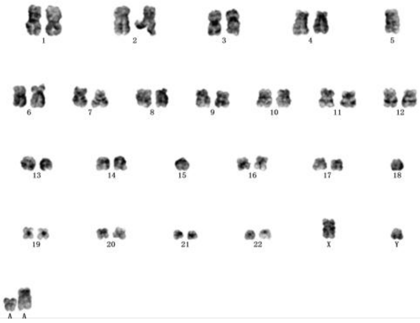

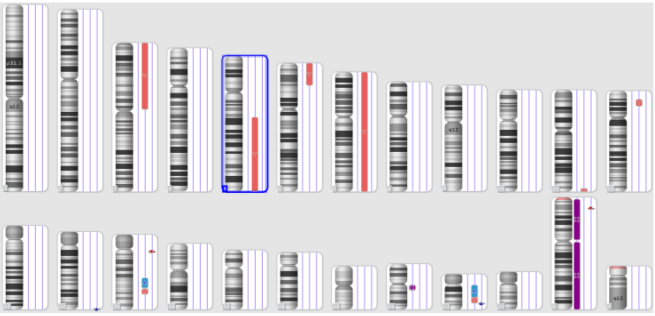

Figure S22.1: Metaphase Cytogenetics indicates 41～45,XY,del(2)(q33),-5,del(7)(p13), add(11)(q23), +2～3mar,inc[cp5]/46,XY[2]

Figure S22.2: SNP-A results indicate complex chromosomal aberrations (Table S22).

Table S22: Chromosomal lesions detected by SNP-A in Case 22#.

Type	Microarray Nomenclature	Size(Kbp)	CN state
LossMosaic	arr[hg19]<br>3p26.3p11.1 (61,891-88,225,600)x1-2	88164	1.6
LossMosaic	arr[hg19]<br>5q14.2q35.3 (82,350,935-180,715,096)x1-2	98364	1.3
LossMosaic	arr[hg19]<br>6p25.3p22.1 (156,974-29,150,160)x1-2	28993	1.6
LossMosaic	arr[hg19]<br>7p22.3q36.3 (43,376-159,056,248)x1-2	159013	1.6
LossMosaic	arr[hg19]<br>12p13.2p12.2(11,751,919-20,263,885)x1-2	8512	1.8
GainMosaic	arr[hg19]<br>15q22.2q23 (59,622,460-72,570,680)x2-3	12948	2.3

LossMosaic	arr[hg19] 15q24.1q25.1(74,256,532-81,075,769)x1-2	6819	1.5
UPD	arr[hg19] 20q11.21q11.23(29,510,306-36,082,411) hmz	6572	2
LossMosaic	arr[hg19] 21q22.11q22.13(31,887,600-39,263,244)x1-2	7376	1.7
GainMosaic	arr[hg19] 21q11.2q22.11(15,016,486-31,882,693)x2-3	16866	2.6

Case 23#，male，28 years old，sAML

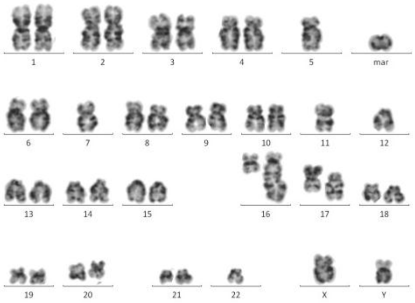
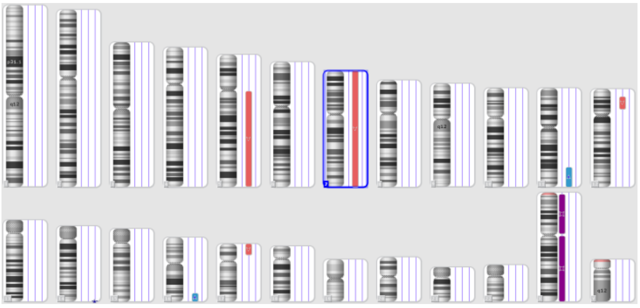

Figure S23.1: Metaphase Cytogenetics indicates 42,X,add(Y)(p11),-5,-7,-11,-12,del(12)(p11),add(16)(q24),add(17)(q25),add(17)(p13), -22,+r[16]/46,XY[4]

Figure S23.2: SNP-A results indicate complex chromosomal aberrations (Table S23).

Table S23: Chromosomal lesions detected bySNP-A in Case 23#.

Type	Microarray Nomenclature	Size(Kbp)	CN state
LossMosaic	arr[hg19] 5q11.1q35.3(50,364,961-180,715,096)x1-2	130350	1.6
LossMosaic	arr[hg19] 7p22.3q36.3(43,376-159,119,707)x1-2	159076	1.6
GainMosaic	arr[hg19] 11q22.3q25(108,340,592-134,937,416)x2-3	26597	2.5
LossMosaic	arr[hg19] 12p13.2p11.23(10,970,781-27,789,635)x1-2	16819	1.7
GainMosaic	arr[hg19] 16q23.1q24.3(78,795,974-90,155,062)x2-3	11359	2.5
LossMosaic	arr[hg19] 17p13.3p12 (525-15,068,104)x1-2	15067	1.7

Case 24#，male，84 years old，sAML

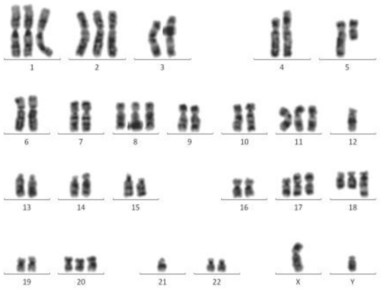

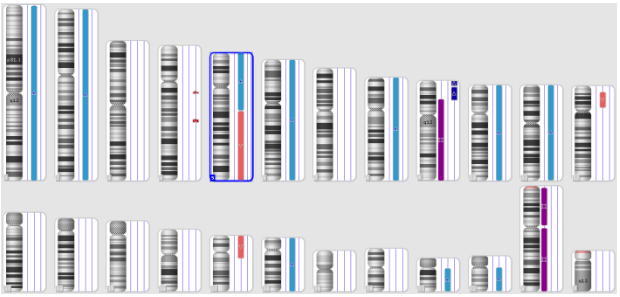

Figure S24.1: Metaphase Cytogenetics indicates 54～56,XY,+1,+2,der(4;12)(q10;q10),+5,del(5)(q13q31)×2, add(7)(q32),+8,+11,del(12)(p11), +17,i(17)(q10)×2,+add(18)(q23),+21,+22[cp18]/46,XY[2]

Figure S24.2: SNP-A results indicate complex chromosomal aberrations (Table S24).

Table S24: Chromosomal lesions detected by SNP-A in Case 24#.

Type	Microarray Nomenclature	Size(Kbp)	CN state
GainMosaic	arr[hg19] 1p36.33q44(849,466-249,224,684)x2-3	248375	2.7
GainMosaic	arr[hg19] 2p25.3q37.3(12,770-242,782,258)x2-3	242769	2.6
GainMosaic	arr[hg19] 5p15.33q14.2(113,576-81,475,075)x2-3	81361	2.6
LossMosaic	arr[hg19] 5q14.3q35.3(83,005,173-180,715,096)x1-2	97710	1.2
GainMosaic	arr[hg19] 6p25.3q27(156,974-170,914,297)x2-3	170757	2.7
GainMosaic	arr[hg19] 8p23.3q24.3(158,048-146,295,771)x2-3	146138	2.7
Gain	arr[hg19] 9p23p21.2(9,027,217-26,610,103)x3	17583	3
UPD	arr[hg19] 9p21.2q34.3(25,953,109-141,011,581) hmz	115058	2
GainMosaic	arr[hg19] 10p15.3q26.3(100,047-135,426,386)x2-3	135326	2.6
GainMosaic	arr[hg19] 11p15.5q25(230,680-134,937,416)x2-3	134707	2.7
LossMosaic	arr[hg19] 12p13.31p11.21(9,228,433-30,790,954)x1-2	21562	1.4
LossMosaic	arr[hg19] 17p13.3q12(525-32,850,419)x1-2	32849	1.3
GainMosaic	arr[hg19] 18p11.32q23(136,227-78,013,728)x2-3	77877	2.6
GainMosaic	arr[hg19] 21q11.2q22.3(15,016,486-48,093,361)x2-3	33076	2.7

GainMosaic	arr[hg19] 22q11.1q13.33(16,888,899-51,197,766)x2-3	34308	2.7

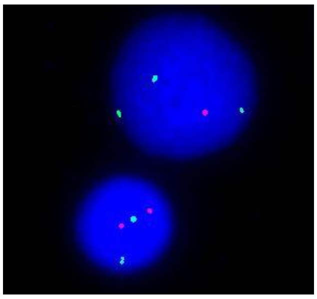

D5S23, D5S721/CSF1R

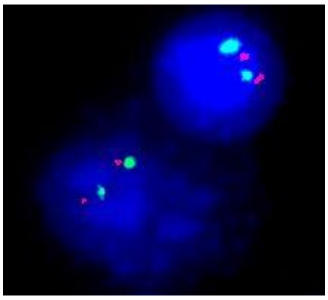

D7Z1/D7S486

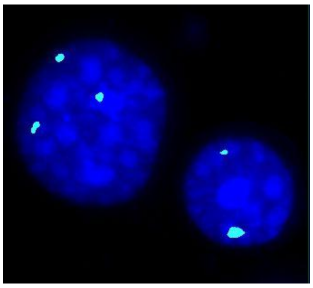

D8Z2

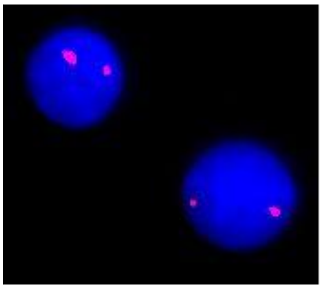

D20S108

FISH indicates：

nuc ish(D5S23,D5S721×3),(CSF1R×1)[152/400]

nuc ish(D7Z1×2),(D7S486×2)[400]

nuc ish(D8Z2×3)[120/400]

nuc ish(D20S108×2)[400]

Case 25#，female，53 years old，sAML

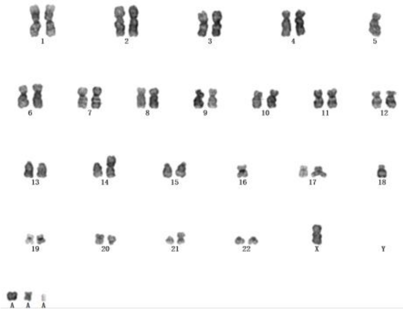

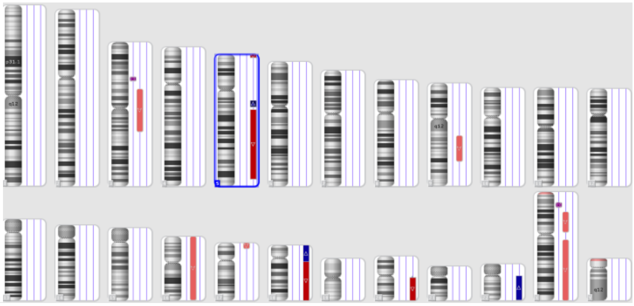

Figure S25.1: Metaphase Cytogenetics indicates 41～44,X,-X,-5,add(14)(p11),-16,-18, ?del(20)(q11),add(21)(p11),+r,+mar,1dmin[cp9]/46,XX[1]

Figure S25.2: SNP-A results indicate additional chromosomal aberrations (Table S25).

Table S25: Chromosomal lesions detected by SNP-A in Case 25#.

Type	Microarray Nomenclature	Size(Kbp)	CN state
LossMosaic	arr[hg19] 3p14.1q21.1(64,586,466-122,786,085)x1-2	58200	1.7
UPD	arr[hg19] 3p21.31p21.1(47,375,954-52,595,417) hmz	5219	2
Loss	arr[hg19] 5q13.3q35.1(75,996,797-171,332,938)x1	95336	1
Gain	arr[hg19] 5q12.3q13.2(63,525,704-71,906,934)x4	8381	4
LossMosaic	arr[hg19] 9q21.11q31.1(72,082,004-106,632,551)x1-2	34551	1.8
LossMosaic	arr[hg19] 16p13.3q24.3(85,880-90,155,062)x1-2	90069	1.1
LossMosaic	arr[hg19] 17p13.3p13.1(525-7,533,753)x1-2	7533	1.2
Gain	arr[hg19] 18p11.32q11.2(136,227-22,955,019)x3	22818	3
Loss	arr[hg19] 18q11.2q23(22,956,086-78,013,728)x1	55057	1
Loss	arr[hg19] 20q11.21q13.33(30,882,070-62,913,645)x1	32031	1
Gain	arr[hg19] 22q11.1q13.33(16,888,899-51,107,063)x3	34218	3
LossMosaic	arr[hg19] Xp21.3p11.21(28,447,571-56,696,092)x1-2	28248	1.7
LossMosaic	arr[hg19] Xq13.1q28(68,576,808-155,233,098)x1-2	86656	1.6
